# Irregular practices in drugstores in the offer of products for children under three years old

**DOI:** 10.1590/1984-0462/2024/42/2022177

**Published:** 2023-10-23

**Authors:** Roberta Almeida Silva Soares, Elisa Natany Silva Pinheiro, Maria Hortência Teixeira Diniz, Ana Paula Bortoletto Martins, Antônio Marcos Machado de Oliveira, Leandro Alves Pereira, Luciana Saraiva da Silva, Ana Elisa Madalena Rinaldi

**Affiliations:** a Universidade Federal de Uberlândia, Uberlândia, MG, Brazil.; b Universidade de São Paulo, São Paulo, SP, Brazil.

**Keywords:** Infant formula, Products commerce, Nursing bottles, Sanitary supervision, Fórmulas infantis, Comercialização de produtos, Mamadeiras, Fiscalização sanitária

## Abstract

**Objective:**

To analyze the compliance with the commercialization of children's products included in the Brazilian Code of Marketing of Infant and Toddlers Food and Childcare-Related Products (NBCAL) in drugstores in Uberlândia/MG.

**Methods:**

A cross-sectional study was carried out in 143 drugstores that sold infant products: infant formula (IF), follow-up IF, nipples, teats, pacifiers and nipple shields; FI for young children, transition foods and cereal-based foods, fluid or powdered milk, modified/similar milks of plant origin and dairy compounds. The location of drugstores in the five geographic sectors was performed by geoprocessing. The data collected were: types of promotion and types of drugstore administration (drugstore chains/drugstores with independent administration). Irregular commercial promotion was expressed as absolute and relative frequencies.

**Results:**

Irregular commercial promotion was found in 11.7% of nipples, pacifiers and bottles, in 10.0% of IF and follow-up formula, in 9.5% of IF for young children, in 11.1% fluid or powdered milk, in 25.0% of transition foods and cereal-based foods and in 59.1% of dairy compounds. In commercial drugstore chains, the presence of promotion for dairy (81.8 vs. 28.6%, respectively) was higher than in drugstores with independent administration. The opposite ocurred for fluid or powdered milk, modified and similar milks of plant origin. The downtown and eastern sectors had the highest percentages of promotions (26%).

**Conclusions:**

NBCAL violations still occur in drugstores, mainly in the sale of young children's foods and in the commercial network drugstores.

## RESUMO

**Objetivo:**

Analisar a conformidade da comercialização dos produtos infantis incluídos na Norma Brasileira de Comercialização de Alimentos para Lactentes e Crianças de Primeira Infância, Bicos, Chupetas e Mamadeira (NBCAL) e de compostos lácteos em drogarias de Uberlândia/MG.

**Métodos:**

Estudo transversal realizado em 143 drogarias que vendiam produtos infantis: fórmulas infantis (FI) para lactentes, FI de seguimento para lactentes, mamadeiras, bicos, chupetas e protetores de mamilo; FI para crianças de primeira infância, alimentos de transição e alimentos à base de cereais, leites fluidos/em pó, leites modificados/similares de origem vegetal e composto lácteo. A localização das drogarias nos cinco setores geográficos foi realizada por geoprocessamento. Os dados coletados foram: tipos de promoção comercial irregular e tipo de administração da drogaria (rede/independente). As promoções comerciais irregulares foram expressas em frequências absoluta e relativa.

**Resultados:**

Verificamos a presença de promoção comercial irregular em 11,7% dos bicos, chupetas e mamadeiras, em 10,0% das FI lactentes/seguimento de lactentes, em 9,5% das FI para crianças de primeira infância, em 11,1% dos leites, em 25,0% de alimentos de transição e em 59,1% dos compostos lácteos. Nas drogarias de rede, a presença de promoção comercial irregular foi maior para compostos lácteos (81,8 *vs.* 28,6%, respectivamente) e, para leites, foi maior nas drogarias independentes (30,8 *vs.* 6,0%). Os setores central e leste apresentaram os maiores percentuais de promoção comercial irregular (26%).

**Conclusões:**

As violações à NBCAL ainda ocorrem nas drogarias, principalmente para os produtos destinados às crianças de primeira infância, e nas drogarias de rede.

## INTRODUCTION

Adhesion to and the status of the International Code of Marketing of Breast-Milk Substitute^1^ is different among countries. The recent Code Status Report^2^ provided data from 194 countries and verified the adoption of new strategies to restrict the marketing of infant and toddler's foods. In 70% of countries there is some form of legal measure in full force, or with at least some of the provisions from the code applied. The other 30% did not adopt any marketing restriction. The main justifications for the illegal marketing practices were lack of interest in the code, lack of responsibility of health agencies, absence or low frequency of monitoring, lack of understanding of the Code, and low human and financial resources.^2^

In Brazil, the first version of the code was created as the “Regulation for the Commercialization of Infant Foods” (*Norma para Comercialização de Alimentos para Lactentes* — NCAL)^3^ and has since moved from the status of regulation to resolution^4,5^ and later to law,^6,7^ in addition to having increased the number and type of products to be inspected.

Commercial promotion is defined as a set of informational and persuasive activities — originating from companies responsible for the production, manipulation, distribution, or marketing of the products encompassed by the decree in question — which includes promotions by means of sounds, images, or a combination thereof, with the aim of inducing the acquisition or purchase of a particular product.^7^ Article 5 of Decree No. 9.579/2018^7^ prohibits commercial promotion for infant formulas, follow-up formulas, formulas for high-risk babies, bottles, nipples and pacifiers. Article 6 reports that commercial promotion is allowed for toddler formula; complementary and cereal-based foods; liquid/powdered, modified, or plant-based milk, as long as these products include specific label information or auditory elements recommended by the Ministry of Health.

In 2013, the International Baby Food Action Network (IBFAN) monitored compliance with the NBCAL in 12 Brazilian municipalities. Non-compliance with Law 11,265/2006^6^ was verified in several establishments, totalizing 65 irregularities, the main ones being found in promotional materials, product labels, and publications of educational materials. From a total of 49 companies, only 18 responded to notifications sent by IBFAN. From 2007 to 2014, IBFAN identified an increase in the promotion and advertising of infant formula.^8^

A study conducted in the city of Rio de Janeiro including infant products whose commercial promotion is illegal (infant formulas, follow-up formulas, formulas for high-risk babies, bottles, nipples and pacifiers) found that 20.3% of the surveyed establishments did not comply with the legislation.^9^ Another study that considered all products, including toddler formula; complementary and cereal-based foods; liquid/powdered, modified, or plant-based milk found that 62.8% of establishments violated NBCAL.^10^ In both studies,^9,10^ the most frequent commercial promotion strategies were discounts and special exposures.

There have been significant advances of the NBCAL in Brazil, from its approval as a law to the substantial change in product labels and the range of products. One of the biggest challenges is the monitoring of law abidance at the national level conducted by the Brazilian Health Regulatory System and, consequently, the fining of companies that violate it. Periodic monitoring has been conducted by IBFAN^8^ and can be further complemented by regional studies to reach the national situation. Monitoring is relevant for the application of fines. Thus, this study aimed to analyze compliance with legislation in the commercialization of the products included in NBCAL in drugstores in Uberlândia, state of Minas Gerais, Brazil.

## METHOD

This was a cross-sectional study, conducted in drugstores of Uberlândia, state of Minas Gerais, in 2019. The sample was selected from the 322 drugstores that had their addresses listed and made available by the Municipal Health Regulatory Agency. The sample was estimated by simple random sampling proportional to the number of drugstores in each neighborhood within the five geographic sectors (north, south, downtown, east, and west), considering a maximum error of 5% and a confidence level of 95%. The study variables were categorical, and since there were no historical data in the municipality, a conservative calculation of the sample size was adopted to estimate proportions.

The initial sample consisted of 194 drugstores: 50 (25.8%) were not found in the corresponding addresses and one (0.5%) did not consent to the research. At least one drugstore from every neighborhood of each commercial network was visited.

Locating the drugstores in each neighborhood and geographic sectoring were performed through the spatialization of all drugstores via geocoding in Google Maps, which consists of creating a Keyhole Markup Language (KML) file by importing an address sheet elaborated in Excel. Subsequently, based on the KML file, a Shapefile layer (SHP) was created using the Geographic Information System (QGIS). This layer was overlaid with the shapefile of the urban area divided by sectors and based on Google Road, available in QuickMap Services. The final map was generated in Print Composer.

A form was prepared to verify which infant and toddler's foods were sold and the presence or absence of irregular commercial promotions, based on the “Training Course Manual for Code Monitoring” (*Manual de Curso e Capacitação em Monitoramento*) (IBFAN BRASIL). The products included in this form were: infant formula, follow-up formula, baby bottles, bottle nipples, pacifiers, and nipple protectors — for which sales advertisements is prohibited; toddler formula, complementary and cereal-based foods, and liquid/powdered, modified or plant-based milks — for which sales advertisements are allowed if the recommended visual elements are followed; and dairy compounds — whose commercial promotion was not regulated in NBCAL, but in this study was considered irregular if there were no recommendations made by the Ministry of Health.

This form listed all the baby products available in the drugstore and the presence of sales advertisements (no/yes). The form was completed with direct observation by the authors of the study. The five types of commercial promotion analyzed were: special exhibition, discount coupons, posters, special offers, and linked sales or gifts. The form also included data on the drugstore's administration type — drugstore chains and drugstores with independent administration.

The definition of the products to be analyzed followed the description used by Decree 9,579/2018.^7^ This decree does not include regulation of the sale of toddler milk, but we chose to include it in the study since it is a product sold as being similar to infant formula. This product is defined by Normative Instruction No. 28^11^ as a powdered product resulting from the mixture of milk and dairy or non-dairy food product(s) or substance(s), or both, which is suitable for human consumption and obtained by a technologically appropriate process. Dairy ingredients must represent at least 51% mass/mass (m/m) of the total ingredients (required or raw material) of the product. The outcomes in our study were the irregular commercial promotion of all infant and toddler foods (yes/no).

The data were analyzed using the EpiInfo software version 7.2.3.1 and Stata^®^ software. The results were expressed in absolute and/or relative frequencies for the total number of drugstores, type of administration, type of irregular commercial promotion and geographic sector. The chi-square test was used to verify whether there was a statistically significant difference between the frequency of irregular commercial promotion and the type of administration of the drugstore and geographic sector. The significance level used was 5%.

The study was submitted and approved by the Research Ethics Committee of the Federal University of Uberlândia (Certificate of Presentation for Ethical Consideration — CAAE: 05947018.2.0000.5152).

## RESULTS

**Figure 1:**
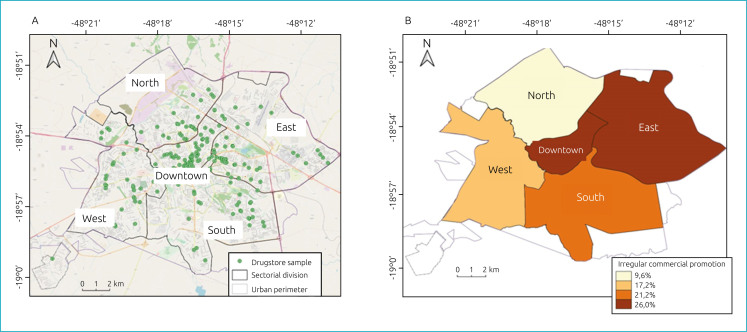
Mapping of drugstores sample (A) and frequency of irregular commercial promotions (B) by geographic sectors. Uberlândia (MG), 2019.

The final sample consisted of 143 drugstores, 17 (11.8%) located in the north, 28 (19.6%) in the south, 35 (24.5%) downtown, 35 (24.5%) in the east, and 28 (19.6%) in the west (Figure 1A). There were 73 (51%) drugstores with independent administration and 70 (49%) drugstore chains.

The infant and follow-up formula were sold in 83.9% of drugstores; pacifiers, nipples, bottles, and nipple protectors in 95.8%; toddler formula in 73.4%; fluid/powdered, modified, and plan-based milks in 44.1%; complementary and cereal-based food products in 50.4%; and dairy compounds in 80.4% of the drugstores (Table 1). Regarding the infant foods and products for which commercial promotion is prohibited, we found irregular commercial promotion in 11.7% of the drugstores for baby bottles, nipples, pacifiers, and nipple protectors. Bottles showed the highest frequency of irregular commercial promotion (51.4%), followed by pacifiers (40.5%), nipple protectors (5.4%) and nipples (2.7%) (data not showed in tables and figures). Irregular commercial promotion of infant formula and follow-up formula was found in 10% of the drugstores that sold them (Table 1). In view of the mandatory label information recommended by the Ministry of Health, which must be presented with the products that are allowed to have commercial promotion, irregular commercial promotion was verified in 9.5% of the toddler formulas, 11.1% of liquid/powdered, modified, and plant-based milks, 25% of baby foods, and 59.1% of dairy compounds (Table 1).

**Table 1:** Frequency of drugstores that sold infant and toddler foods and products and that practiced irregular commercial promotion. Uberlândia (MG), 2019.

	Drugstore	
	Total that sold n (%)	Practiced irregular commercial promotion n (%)
Infant formula and follow-up formula	120 (83.9)	12 (10.0)
Baby bottles, nipples, pacifiers, and nipple protectors	137 (95.8)	16 (11.7)
Toddler formula	105 (73.4)	10 (9.5)
Liquid/powdered, modified or plant-based milks	63 (44.1)	7 (11.1)
Complementary and cereal-based food	72 (50.4)	18 (25.0)
Dairy compounds	115 (80.4)	68 (59.1)

The presence of irregular commercial promotion was higher in drugstore chains for dairy compounds, and higher for liquid/powdered, modified or plant-based milks in drugstores with independent administration (Table 2). The type of irregular commercial promotion most found for the products analyzed was special exposure, followed by special offers, for both the products that are prohibited to have commercial promotion and for those that are allowed. Dairy compounds were the products that presented five types of irregular commercial promotion (Table 3).

**Table 2:** Frequency of drugstores that sold infant and toddler foods and product and that practiced irregular commercial promotion by type of administration of the drugstore. Uberlândia (MG), 2019.

	Drugstores with independent administration			Drugstore chains			p-value
	Total that sold	Practiced irregular commercial promotion		Total that sold	Irregular commercial promotion		
	n	n	%	n	n	%	
Infant formula and follow-up formula	53	5	9.4	67	7	10.5	0.854
Baby bottles, nipples, pacifiers, and nipple protectors	68	9	13.2	69	7	10.1	0.573
Toddler formula	41	5	12.2	64	5	7.8	0.440
Liquid/powdered, modified or plant-based milks	13	4	30.8	50	3	6.0	0.011
Complementary and cereal-based food	22	7	31.8	50	11	22.0	0.375
Dairy compounds	49	14	28.6	66	54	81.8	<0.001

**Table 3:** Number of products that had irregular commercial promotion by type of irregular commercial promotion. Uberlândia (MG), 2019.

Products	Type of irregular commercial promotion (n)					Total (n)
	Special exhibition	Poster	Discount coupons	Special offers	Linked sales or gifts	
Baby bottles, pacifiers, nipple protectors and nipples	35	0	0	3	0	38
Baby bottles	18	0	0	1	0	19
Pacifiers	14	0	0	2	0	16
Nipple protectors	2	0	0	0	0	2
Nipples	1	0	0	0	0	1
Infant formula and follow-up formula	17	1	0	6	0	24
Toddler formula	10	1	0	2	1	14
Liquid/powdered, modified or plant-based milks	4	1	0	1	0	6
Complementary and cereal-based food	15	0	1	7	0	23
Dairy compounds	102	6	5	58	13	184

**Figure 2:**
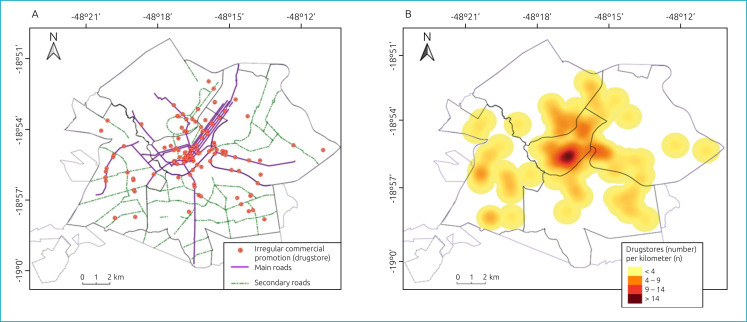
Irregular commercial promotions in drugstores by type of road (A) and number of drugstores per kilometer (B). Uberlândia (MG), 2019.

In the downtown and eastern sectors, higher percentages of irregular commercial promotion were observed for products (Figure 1B). In the downtown and eastern sectors, we found a higher percentage of irregular commercial promotion for bottle nipples, pacifiers, bottles, nipple protectors, infant formula, dairy compounds, and plant-based milks; and in the south and east sectors we found a higher percentage of advertisements for baby foods. The number of advertisements is higher in the main roads of the city and in the central region (Figures 2A and 2B).

## DISCUSSION

Irregular commercial promotion was found for all categories of infant and toddler products analyzed in this study, characterizing irregular, inadequate practices according to current legislation. Dairy compounds, the only products whose mandatory label information was not defined by NBCAL, was the product with the highest percentage of advertisements and the greatest diversity of sales advertisements. The geographic sectors with the highest percentage of irregular commercial promotions for most products were the downtown and eastern ones, with the most frequent types of commercial promotion being special exposure and special offer. We also observed higher irregular commercial promotion for dairy compounds in drugstore chains when compared with drugstores with independent administration.

The frequency of irregular commercial promotion for baby foods was higher than for infant and follow-up formulas. This result was expected, especially since the commercial promotion for baby food is allowed, according to the NBCAL. We highlight, however, that irregular commercial promotion was high for dairy compounds (59.1%), followed by baby food (25%). A study conducted in Teresina, in the state of Piauí,^12^ found that in 29% of irregular commercial promotions for toddler formula, in all establishments, lacked the notice required by the NBCAL. In the United States, there is concern about the increasing sale and advertising of these foods aimed at infants older than 12 months, especially due to the lack of regulation, the use of cross-promotion strategies and of labels similar to infant formula, and due to their prescription by health professionals.^13,14^ The World Health Assembly's resolution 69.9^15^ recommends ending of commercial promotion for products intended for children aged 12 months or older, as well as the adjustment of packaging and labeling, in order to avoid cross-sale with infant formulas.

In our study, the presence of sales advertisements of most types was high for dairy compounds, being more prominent in drugstore chains, in which the sales are defined by the head office. The Ministry of Agriculture, Livestock, and Supply (MAPA)^11^ established the regulation for the identity and quality of these products in 2007, the year following the approval of Law No. 11,265/2006.^6^ Thus, the NBCAL does not provide any regulation for commercialization. One of the strategies used by the companies producing the compounds is having similar packaging and labels to the infant formulas, which can confuse consumers at the time of purchase, inducing them to buy the product.^16^

We found that the violation of NBCAL occurred for all product categories. Our results are in line with those of the monitoring carried out by IBFAN^8^ in previous years and a study conducted in the south of Rio de Janeiro.^9,10^ In studies carried out in Cambodia, Nepal, Senegal, and Tanzania there was also a presence of sales advertisements, and it was less prevalent in countries where the legislation protecting breastfeeding is stronger.^17^ In Zambia, the rate of non-compliance with the code by the manufacturers and distributors of breast-milk substitutes was 8 and 14% respectively; and the main strategy used was special exhibition.^18^ In Piracicaba, in the state of São Paulo, two thirds of the drugstores and half of the supermarkets used special exposure as a marketing strategy, and as an auxiliary strategy they used special offers and gifts.^19^

Our study found a greater number of promotions in the eastern and central regions of the city and in the main streets, which may be directly related to the greater movement of people, due to the high concentration of commercial establishments present there. In the eastern sector, the presence of attractive establishments stands out, such as shopping malls, the Municipal Administrative Center, the Federal University of Uberlândia, sports parks and stadiums and large companies that operate in the agri-food, telecommunications and entertainment sectors, in addition to the region's main public hospital, the Clinical Hospital of the Federal University of Uberlândia and the Cancer Hospital.^20,21^ In addition to a wide shopping complex, the central sector has the highest population density.^20^

In the worldwide review of the status of the World Health Organization (WHO) Code,^2^ only three countries achieved scores above 90, but none reached 100 (maximum score), an indication that all countries need to adjust their laws. Brazil scored 83 points, being classified as a country aligned with the Code. The category with the lowest score was the one that concerns the commitment of legislation by workers and the health system, which reinforces the need to expand the knowledge/training of employees and supervision by the municipal and state health surveillance. In Brazil, the Brazilian Health Regulatory System is responsible for surveilling products and services of interest to health — a very wide volume of activities and attributions. One of the difficulties with the supervision of NBCAL products is the lack of structure and employees to fulfill all the duties. Although the health regulatory agencies are responsible for monitoring, we emphasize the important role of healthcare professionals in knowing and respecting the NBCAL, and assisting with the identification and reporting of violations. Moreover, the discussion about the insertion of this theme should be addressed more specifically in the curricula of undergraduate courses. IBFAN plays an important role in identifying violations, especially via constant monitoring, training of health professionals, and educational activities on the subject.

The factors that may explain the irregularities and inappropriate practices found in this study, as well in the previous ones, are lack of supervision by the competent authorities and, consequently, lack of punitive action toward companies, low knowledge of the legislation by employees of commercial establishments, commercial relations between establishments and the companies aiming at increasing sales with sales advertisements; in addition to the aggressive investment of companies in marketing strategies and their influence on baby product companies, which have an important market share worldwide, making billions from the sale of products using marketing strategies.^22^ The sale of children's products is controlled by a few multinational companies, adopting very similar marketing strategies in different countries. The sale of these products increases according to a country's income, its rate of urbanization, medicalization of food (companies create specific products for children older than one year), maternal work (infant formulas as a substitute for breast milk, allowing women to return to work).^22,23^

A study conducted in the United States in 2020 showed that corporate political activity in the food industry is an important barrier to the development and implementation of public health policies. Companies influence public policy and research. Moreover, they also invest in their direct relationship with the community, media, health professionals, and government representatives, with the intention of passing an impression of legitimacy and trust to the general population.^22,24^

Some of the strengths of this study include incorporating all baby products in the same study, using a form elaborated from the material proposed by IBFAN for NBCAL monitoring, and describing the spatial location of irregular commercial promotions. This mapping will serve as a basis for future studies that will allow us to investigate other possible demographic and social factors possibly associated with the presence of irregular commercial promotion. However, our study also has limitations. We highlight the reduced sample of drugstores due to either their addresses not being located or their nonexistence within a reported address. Additionally, the cross-sectional design of the study could not follow long-term marketing practices to verify the duration of the sales advertisements, nor its reasons, the frequency at which they were performed, and if new sales strategies were implemented.

We conclude that violations of NBCAL still occur in drugstores. It is necessary to increase the supervision of the Code, with significant fining for companies and establishments that do not comply with it, in addition to establishing a regulation for the commercialization of dairy compounds since these were the products with the highest percentage of sales advertisements.
